# The action logic of the older adults about health-seeking in South Rural China

**DOI:** 10.1186/s12889-023-17314-y

**Published:** 2023-12-12

**Authors:** Jianqiang Lin, Dan Yang, Xinyu Zhao, Liqiong Xie, Kun Xiong, Lei Hu, Yue Xu, ShanShan Yu, Wenyong Huang, Ni Gong, Xiaoling Liang

**Affiliations:** 1 State Key laboratory of Ophthalmology, Zhongshan Ophthalmic Center, Sun Yat-sen University, Guangzhou, China; 2 School of Nursing, Jinan University, Guangzhou, China; 3 Department of Ophthalmology, the First Affiliated Hospital of Guangzhou Medical University, Guangzhou, China; 4 Department of Endodontics, Stomatological Hospital of Guangzhou Medical University, Guangzhou, China

**Keywords:** Health-seeking behavior, Rural China, Older people, Participatory rural appraisal, In-depth interviews

## Abstract

**Background:**

The Chinese government has invested significant resources to build many rural healthcare stations. However, in the face of convenient medical paths and accessible medical resources, the utilization rate of health services for older adults in rural areas is surprisingly low. This study explored why health-seeking behavior among older adults in rural China was not active.

**Methods:**

Data were collected through participatory rural appraisal (PRA) with 108 participants in 12 villages in southern China. Daily schedule and social and resource mapping were employed to outline the range of activities and the routine of the older adults, as well as in-depth interviews to understand the logic of their healthcare choices. Data collected were analyzed by content analysis.

**Results:**

Three themes were generated: (1) perceptions of health status (being healthy or sick): the rural older adults used the ability to handle routine chores as a measure of health status; (2) prioritization of solving symptoms over curing diseases: the older adults preferred the informal self-medication to cope with diseases, as long as there were no symptoms and no pain; (3) ‘unpredictable’ troubles: they tended to favor the ‘optimal’ solution of keeping their lives in order rather than the best medical treatment options.

**Conclusion:**

This study showed that the medical practices of the rural elderly were profoundly influenced by their perceptions of health and their life experiences. In the face of diseases, they tended to keep their lives in order, preferring self-treatment practices that address symptoms or selectively following medical advice rather than medical and science-based clinical solutions. In the future, the construction of rural health care should focus on changing the ‘inaccessibility’ of healthcare resources at the subjective level of the rural elderly and develop culturally adaptable health education.

**Supplementary Information:**

The online version contains supplementary material available at 10.1186/s12889-023-17314-y.

## Background

As one of the most rapidly aging countries, China is facing a growing health burden [1]. By the end of 2020, the population aged 60 or above in China had reached 264 million, accounting for 18.7% of the total population [2]. More importantly, 60% of older people live in rural areas [3]. It is well-known that chronic diseases have become a major threat to older people in rural China. The prevalence of chronic illness among older people in rural China was 82.6%, with 45.9% of them suffering from two or more chronic illnesses [4, 5]. Chronic disease is the leading cause of death in China, accounting for an estimated 88% of deaths, and its disease burden accounts for about 70% of the total disease burden in China [6]. As a result, the health of older people in rural China has become a major public health challenge.

To address the issue of health equity for the rural older population, the construction of the primary healthcare system in rural areas has been the focus of healthcare reform in China [7]. By 2020, the New Rural Cooperative Medical Scheme (NCMS) had almost achieved universal coverage among rural populations, and healthcare expenditure in China had increased to 1.6797 trillion RMB by 2019, with substantial preference policies for the grassroots, impoverished, and remote areas [8]. Each village has at least one village clinic, which allows 80% of the residents to access primary care within a 15-minute walking distance [9, 10]. Meanwhile, the National Health Commission (NHC) of the People’s Republic of China has formulated a set of free health management plans targeting at the older population [11]. After years of efforts, Chinese healthcare reform has made remarkable advances in achieving population coverage, service coverage, and cost coverage for the health of rural populations, and health resources and access increased by about 50% in rural China [12–14].

Despite these advances, considerable evidence reported that productive health-seeking behaviors were not common in rural China and older patients thus received inappropriate or no treatment [15–17]. Only 37.4% of rural Chinese adults aged 60 or above had an annual physical examination, and less than 30% of patients with hypertension and diabetes were receiving treatment [10, 18]. Besides, most rural Chinese adults managed their illnesses on their own without seeking help from health care [19]. For example, people in the area where this study was conducted often apply herbal medicine or food as a self-medication programme to cope with illnesses [20]. Even though there were already abundant healthcare resources available in rural areas, why didn’t older adults seek the help of healthcare professionals? The use of medical services for older people in rural areas depended on a number of factors. First, the city-oriented economic reform had led to a concentration of health resources in the urban areas, and the time and transportation costs discouraged the rural senior’s use of healthcare services [21]. Second, due to the disparity between rural and urban health insurance, rural older enrollees were subject to more restrictions and financial barriers in terms of benefits and reimbursement rates, resulting in the under-utilization of healthcare services [22–24]. Some studies sought to determine the factors of health service seeking from the perspective of older adults; for example, health literacy affected the health behavior of older people [25]. Illiterates, who made up the majority of older people in rural China, had lower health literacy, poorer self-management skills, and lower use of preventive services [26]. Social demographic characteristics such as gender, age, income, and marital status also affected the utilization of health services [15, 27, 28]. Some quantitative studies examined how rural society and cultural values undermined their healthcare needs [29, 30]. The rural elders were structurally and culturally disadvantaged compared with their urban counterparts: the habitus of rural elders manifested in their evaluation of themselves and their health as inferior and unworthy of treatment, which inhibited their expectations of medical treatment [30]. Nevertheless, these studies were mainly quantitative which did not explain the causal relationships inherent in medical help-seeking among rural older people, and the qualitative study did not focus on the reasons why rural elders still adopted negative medical practices after systematic improvements in rural medical resources.

As one of the methods of participatory research, participatory rural appraisal (PRA) is one approach that empowers participants to generate, analyze and own research data, which has been adapted in many fields such as natural resource management, agriculture, poverty, health care and so on [31–33]. For example, a study from rural India facilitated community engagement of rural stakeholders through PRA, identifying and exploring the contextual factors embedded in the current healthcare delivery process [33]. This study is a creative use of the PRA to assess the health care practice behaviors of older people in rural China. As far as we know, this study is the first study to apply PRA in the healthcare in China. Therefore, in this study, PRA is used to explore the characteristics and decision-making processes of medical practices of rural older people who cope with their medical needs.

## Methods

### Study design

PRA is employed, a social science survey method for analyzing farmers and their behaviors in a rural setting [31]. Through PRA, researchers could build trust with participants and mobilize them to talk about their medical practice behaviors in a short time. Data collection was used by three PRA tools: daily routine, social and resource mapping, and semi-structured interview. Through social and resource mapping, participants presented the scope of activities and types of facilities in their daily life in the form of a simplified map to help the researchers visualize the life circle of the older people. The daily routine was mainly used to collect and analyze the daily activity schedule of the rural older adults so as to understand their production and life. To better understand the characteristics of old people’s access to healthcare in rural area, the researchers encouraged the participants to reflect the facilities and daily arrangements related to their healthcare needs in the social resource mapping and the daily routine. Information from the interview guide and diagrams was used to further explore the behavioral logic of rural older people coping with illness. Social and resource mapping was supposed to be a collective endeavor by residents. However, considering the physical condition of the participants, such as mobility issues, the researchers collected social and resource mapping for each individual separately. The Consolidated Criteria for Reporting Qualitative study checklist (COREQ) was followed (Appendix 1).

### Setting and participants

The study was conducted in a rural area of a city in southern China. The city involves a total registered population of 557,900, of which 436,600 are permanent residents, and is divided into 8 towns and 138 villages [34]. As the medical team to which researcher (JQL) belongs provides healthcare services to 28 villages in the city every year, 12 of these villages were selected for participants recruitment. These villages have a similar geographic location (within 10 km of the city center) and economic development. Participants were recruited through the local village committees, an administrative institution in rural China responsible for the provision of social welfare and social services for the rural older people. With the help of village committees, the researchers visited 12 villages one after another and employed a purposive sampling method. All participants were contacted from researchers via face-to-face invitation. Inclusion criteria were: (1) age of 60 or above (the standard for the elderly classification is 60 years old and above in China); (2) registration as a rural citizen who has lived in the countryside for a long time; (3) clear cognition of the participant and no communication barrier with the researchers; and (4) informed consent and voluntary participation. Exclusion criteria was: people with cognitive impairment or other psychiatric disorders. The PRA was conducted at participant’s home. By the time three villages had been visited, most of the interview information was repeated and the data of interviews had reached the saturation, and the number of participants was 32, 10 in villages A, 12 in villages B, and 10 in villages C. In order to collect more information about the activity resources and daily routine of the rural older adults, the research team visited the other nine villages to recruit more participants, including 8 in villages D ~ G, 10 in village H and K, 6 in village I and J, and 12 in village L. When the 103rd participant was completed, the content of diagrams information was already very rich and most of the information had been repeated. In order to confirm whether the diagrams information was saturated, five additional participants were added for verification.

A total of 108 participants (49 males and 59 females) from 12 villages were included, ranging in age from 60 to 93 years old, with an average age of 74.19 years. All had one or more chronic diseases such as hypertension and arthritis. The duration of the interviews ranged from 20 to 50 min, with an average time of 42.6 min. Demographic characteristics of participants were shown in Table 1.

**Table 1:** Characteristics profile of respondents (n = 108)

Category	Sub - Category	n(%)
**Sex**	Male	49 (45.4)
	Female	59 (54.6)
**Age(years)**	60–74	58 (53.70)
	75–89	43 (39.81)
	90–100	7 (6.48)
**Educational status**	Illiteracy	39 (36.11)
	Primary school	47 (43.52)
	Junior high school	17 (15.74)
	High school	5 (4.63)
**Health condition**	1–2 chronic illness	89
	≥ 3 chronic illness	19

### Data collection

Data collection took place between November 2020 and January 2021. The PRA were conducted face-to-face and completed in the participants’ preferred language – Mandarin, Cantonese, then translated and transcribed into English. The medical anthropologist (NG) first trained the researchers in the use of the PRA tools and interview techniques. The interview guide was developed based on a literature review and expert review by experts with extensive research experience in anthropology and public health [29, 30]. And then the interview guide revised by 2 pre-interviews (Appendix 2) to ensure that the interview guide worked as intended. The pilot interviews were included in the final database. All researchers were divided into two groups of three (one moderator, one recorder and one translator) to collect data including verbal information and non-verbal information. The PRA consisted of two steps: First, participants mapped daily schedule and social and resource mapping to describe the distribution of key resources in their life and their activities at different times of the day. Then, interviews were conducted based on the information from diagrams and interview guide to discuss their perceptions of health services, illness, and health, as well as their solutions to health problems, and causes. The PRA was audio-recorded, then transcribed verbatim and anonymized. And the transcripts were managed and analyzed using NVivo 11. All researchers had no established relationship with participants prior to the study.

### Data analysis

Data analysis was conducted simultaneously with data collection. All recordings were transcribed by a researcher and reviewed by the investigators participated in the PRA. All diagrams were scanned into PDF files, and then the information in the diagrams was summarized and analyzed in Microsoft Excel. The medical anthropologist and the collector of the PRA conferred and summarized the final social and resource mapping and daily routine. The activity locations and average distances in the social and resource mapping were used to describe the participants’ approximate range of activities; daily activity items in the daily schedule were roughly divided into four categories: sleeping, farm work, housework, and leisure to describe the participants’ daily activity schedule. Data analysis of interviews and field records was conducted by content analysis [35]. Two researchers independently conducted text analysis and open coding. The overall impression of the data was grasped by repeatedly listening to the recordings and reading the transcripts. The content of each transcript was then coded from key words and phrases. Similar codes were grouped into categories. New categories were added and adjusted as appropriate for an ongoing, non-linear analysis, and themes were generated. Then, discrepancies were discussed and resolved after a meeting. The final themes were reviewed by all authors to ensure the difference between them.

### Ethical consideration

The study was approved by the Ethics Committee of Zhongshan Eye Center, Sun Yat-sen University (Ethical approval number: 2020KYPJ164). Before conducting data collection, all participants were informed of the purpose and content of the study and provided written informed consent. Participants understood that they could discontinue the study at any time.

### Rigor

To guarantee the rigor and credibility of the data, this study adhered to the criteria proposed by Guba (1989) of credibility, transferability, confirmability, and dependability. To improve credibility, in-depth and lengthy interviews were conducted; and the research team maintained an audit trail of all analytical memos, code books, and recorded meeting and corresponding notes. One of the limitations of qualitative research is the transferability of its results. With the aim of ensuring the transferability of data, all cultural and background characteristics of the participants were explained. In addition, the results were checked by five old people in rural who had not taken part in the research. Dependability was demonstrated through an audit trail of the data collection and analysis processes. To ensure confirmability, some of the interviews and transcripts, along with coding, were provided to three other research colleagues who were specialized in qualitative research and confirmed the accuracy of coding.

## Results

**Fig. 1:**
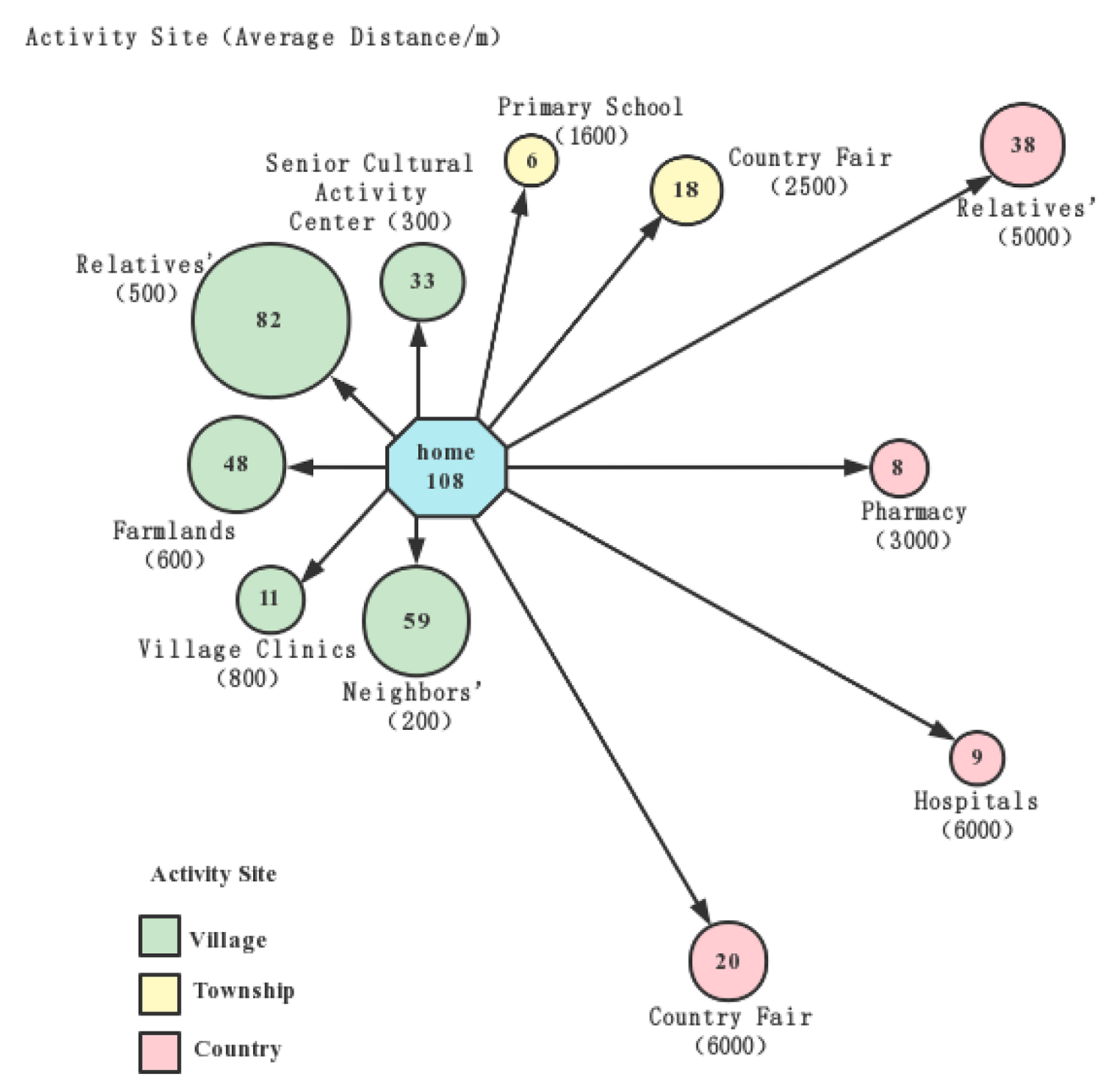
**Summary of Social and Resource Mapping of Rural Senior**: The Fig. 1. describes the range of activities that the rural seniors frequently go, including activity locations and areas. Different colors represent different active areas. The numbers in the circles represent the number of participants to the event site. The length of the line segment from the activity site to the participant’s home represents that the total distance of all the participants to a certain activity site divided by the total number of participants

**Fig. 2:**
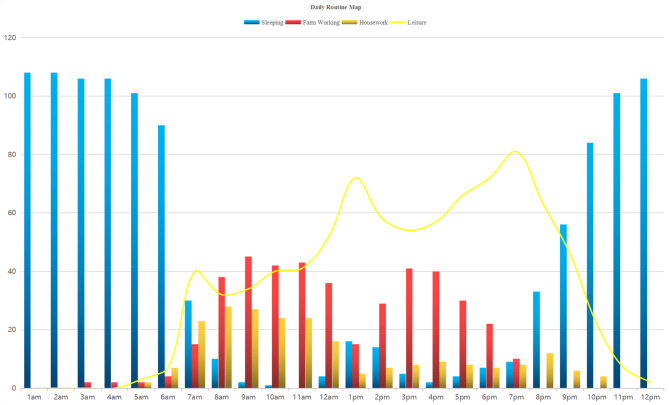
**Summary of the Daily Routine of Rural Senior**: The Fig. 2. summarizes the 24-hour time allocation of all participants. The daily life time allocation of the elderly can be roughly divided into four categories: sleep, farm working, housework, and leisure

Three main themes emerged from the data: (1) perceptions of health status (being healthy or sick); (2) prioritization of solving symptoms over curing diseases; (3) ‘unpredictable’ troubles. Our results found that although health services had been greatly improved in rural China, including facilitated healthcare pathways, improved accessibility to health resources, and expanded coverage of medical insurance, the healthcare utilization of older adults remained low. At the beginning of our study, majority of the participants responded that engagements with many activities and errands hindered health-seeking behavior. However, the social and resource mapping reflected a limited scope of activities of older adults, with most confined within the village and only a few to village clinics and pharmacies for health management (Fig. 1; Table 2). The daily routine and interviews showed that most older adults did not depend on farming for a living and they enjoyed a lot of spare time, and time spent on recreational activities was substantially longer than on farming or housekeeping (Fig. 2). Because of suffering from blurry vision, diminished hearing, arthritis, and other chronic diseases, the older adults had a drab leisure life with low social interaction, such as staying at home or strolling purposelessly at village. Obviously, lacking the time was not the crux of refusing medical service uptake. In fact, the responses from the participants revealed that they had healthcare needs, and had sufficient spare time to make use of the healthcare resources, but their attitudes toward seeking medical care were negative. Our results showed that their utilization of healthcare services had been predominantly influenced by their perceptions of health status, prioritization of solving symptoms rather than curing diseases, and concerns about ‘unexpected’ troubles.

**Table 2:** Summary of Activity Site of Rural Senior

Geographic range of activity	Activity site	Person times
**village**	relatives’	82
	neighbors’	59
	farmlands	48
	senior cultural activity center	33
	village clinics	11
**Township**	country fair	18
	Primary school	6
**county**	relatives’	38
	country fair	20
	hospitals	9
	pharmacy	8

### Perceptions of health status (being healthy or sick)

While sufficient time and healthcare services were conveniently available, the older adults in rural were still reluctant to seek health service. The key reason for this phenomenon was how they perceived health and disease, which would heavily influence their behavioral logic and uptake of medical services. When asked about the definition of health, they used whether and how much they were still able to handle routine chores as measure of health status. They considered that ‘being able to work and take care of themselves’ is healthy, and thus healthcare-seeking was not necessary.

*‘I am healthy if I am not in pain, and I can eat well, sleep well, do my farm work, and take care of myself.’ (R 37, 80, female, hypertension, coronary heart disease, migraine, gastritis)*.

*‘I don’t have any disease, so I don’t need any heath checkup; farmers are not that fragile and precious.’ (R 34, 78, female, hypertension)*.

Even if sickness did exist, they preferred to justify the inaction on healthcare seeking by ‘normalizing’ the discomfort, attributing it to old age and infirmity, and an inevitable stage that all living beings would go through.

*‘I do feel pain in my waist and my legs, and feel dizzy … it is because I am old now, and it is natural to feel unwell from time to time. I am struggling to live out my days, and I am not planning to get them cured.’ (R 36, 70, female, rheumatoid arthritis, lumbar disc herniation, hypertension)*.

Even when obvious symptoms showed up, most participants had difficulty relating the symptoms with any diseases until they produced devastating impacts on their daily life. Under such circumstances, they tended to think that all they needed was to bear with the pains for a few days.

*‘I just had a cataract in my right eye, and my left eye is completely fine. I still can see things and my life is not affected. It does not need any management.’ (R 13, 95, male, cataract, hypertension)*.

*‘The doctor said I had a cataract? So many old people around me had the same problem? It’s not a disease, it happened in old age.’ (R 21, 75, male, hypertension, cataract, rheumatoid arthritis)*.

The rural older adults considered their physical discomfort as a ‘normal phenomenon’ due to aging. Only if their daily performance and life were affected by immobilizing diseases, healthcare-seeking was necessary.

### Prioritization of solving symptoms over curing diseases

When diseases affected the rural older adults’ daily performance, they were more likely to take the experience-oriented self-treatment strategies in the first place. Influenced by the traditional culture and closed social environment, rural older people gave little weight doctors’ advice, and adopted a variety of informal self-treating that have no clinical basis.

*‘I will buy herbal medicine if I don’t feel well. The older generation also drank this medicine, which works better than what the doctor prescribes and is cheaper.’ (R 20, 70, male, hypertension)*.

*‘Coughs and colds are just Shanghuo(heatiness), so I make some herbal tea to ease the symptoms, as all Cantonese do.’ (R 61, 60, female, diabetes, chronic bronchitis)*.

*‘I have unstable blood pressure. I might not be healthy, but my life is not affected; I feel dizzy occasionally, but drinking some water with salt will help. I also heard that when feeling uncomfortable, taking medications that help enrich the blood or pig brain can help strengthen the body.’ (R 34, 78, female, hypertension)*.

Some participants reported unsupervised self-treatment with over-the-counter medications from pharmacies. They believed that buying medicines was an efficient alternative to seeking care from a physician.

*‘When I have pain, like a sore throat, a sore leg, I buy anti-inflammatory drugs, painkillers. Just eat a little, and it’ll be fine.’ (R 25, 66, female, cervical spondylosis, lumbar disc herniation, chronic pharyngolaryngitis)*.

When informal self-treating failed to alleviate or banish the symptoms, the older adults might go to the hospitals for medical treatment. They interacted with medical systems in a manner that we call ‘selective compliance’: they would more likely select those that were most convenient and had the least bearing on their life among all medical advice.

*‘The doctor said that I had lumbar disc herniation and that I needed to go to Guangzhou City for surgery. But I didn’t have the surgery because I did not know any doctors in Guangzhou. I think it is fine just to have analgesic injections when I feel the pain.’ (R 18, 65, male, lumbar disc herniation)*.

They tended to the treatment that downplayed the symptoms of the diseases and took the least time to get themselves back to their routine life rather than highest quality treatment most likely to cure the disease or extend their lives.

*‘I have had the pain in my back for years. I did visit the doctors, but I did not get cured. Now I don’t want to go to the hospital any more. All I can do is to rub some medicinal liquor on the back to release the pain.’ (R 39, 63, female, lumbar strain)*.

The interviews with the older adults indicated that they tended to adopt treatment strategies that banished the symptoms caused by the diseases, rather than the diseases themselves. They considered full compliance to doctors’ advice would bring a lot of trouble to their routine life. When simple and straightforward options were able to alleviate or banish the symptoms and get the problems solved, there was no need for extra efforts and time to cure the disease itself.

### ‘Unpredictable’ troubles

The older adults in rural areas had been accustomed to the established life pattern, and they had an instinctive rejection of everything that affects their daily routine and lifestyle. Despite the willingness to seek healthcare services, the actual health-seeking behavior would change their life patterns and bring about the sense of uncertainty, which curbed their impulse to use medical services. When asked if they would proactively make use of the free healthcare services, the predominant responses were negative. They were concerned that blood tests would do harm to their health, and extra intake of nutritious food was required for recovery. Once a disease was diagnosed, the follow-up treatments for this disease was not free, the healthcare service only identified problems but did not solve them.

*‘I did not go for regular medical check-ups because I didn’t want to have a blood drawn. We have blood draws for examinations when sick or for a regular check-up. My body will get weak, and I would have to eat a lot of chicken to recover from that weakness.’ (R 10, 84, female, lumbar keyboard herniation)*.

*‘For me, regular checkups won’t help too much. I have not prescribed any medication after the checkup, and when it does identify some illness, I will have to go to large hospitals for treatment. I would say I’d better have no checkup at all; out of sight, out of mind’. (R 37, 80, female, hypertension, coronary heart disease, migraine, gastritis)*

Even when a large part of treatment cost was covered by health insurance or even free treatment was available for certain diseases (such as age-related cataract surgery), the extra costs incurred during healthcare-seeking were perceived as barriers, including financial costs (transportation and living expenses), time cost (failing to take care of family members or perform farm work), and so on.

*‘I am too old, so I don’t want to go to the hospital. It is very troublesome and time-consuming there, and the queues are very long. It might take me a whole day just to get an examination done.’ (R 37, 80, female, hypertension, coronary heart disease, migraine, gastritis)*.

*‘My wife is now living in the city to take care of our grandchildren. If I have the surgery done (cataract), my wife will have to come back here to take care of me and won’t be able to help look after the kids.’ (R 29, 70, male, hypertension, cataract)*.

In addition, the rehabilitation cycle was an important factor shaping healthcare decisions. They considered the troubles brought by rehabilitation went beyond their control. They feared that traveling regularly to hospital for rehabilitation took the amount of time and money, which greatly affected their daily labor and their ability to take care for their families.

*‘I can put up with the pain in the leg; it will wear off eventually. Once I take the surgery, no one will take care of me or look after my cows. And they are the only thing I can count on to make some money.’ (R 71, 85, male, hypertension, rheumatoid arthritis)*.

Although the rural older adults could anticipate the troubles caused by seeking medical help, the loss of time and money caused by these troubles was incalculable and even beyond their expectations. In their view, the impact of these troubles was ‘unpredictable’.

## Discussion

This study explained the action logic of rural older adults’ reluctance or even refusal to use health services from the perspective of them. Compared to previous qualitative studies, this study empowered rural older adults through PRA to participate more meaningfully in primary care and contribute to the research. There was enough evidence existing with regard to the effectiveness of the PRA in bringing improvement in the health status of the community [33]. The data obtained from the social and resource mapping showed that the average distance between the primary healthcare facility and the participants’ homes was 800 m, which conformed to the ‘15-minute life circle’ plan, and reflected the convenience and availability of health-care services in the rural areas. However, influenced by the ‘health perspective’ and local culture, the rural older adults with health problems first chose self-treatment and then turned to healthcare services. When faced with medical advice, they tended to selectively follow medical advice to avoid extra trouble to their lives. Therefore, even though rural areas have achieved access to health care resources in terms of time, space, and cost, there is no focus on the subjective level of accessibility for the older adults, resulting in the ‘non-existence’ of plentiful healthcare resources in rural China.

### Health perception featured by the absence of symptoms

In our study, the perception of health referred to the subject’s understanding of health, illness, and death [36]. Compared to the health standards in modern medical science, the criteria to assess health state among older adults in rural China were highly ‘localized’ [37]. In the modern medicine model, the assessment of health is based on results from relevant examinations. Nevertheless, influenced by the life experience of lifelong labor and rural cultures, the older adults defined health by the ability to perform daily work. Self-perceived health state was attached much higher significance than the diagnosis based on objective examination. The result of this study demonstrated that the definition of health was being able to work and losing the ability to perform farm work was regarded as the threshold value for healthcare needs, which was consistent with the findings from some international studies [38–40]. While a study from Chicago found that older people had a holistic conceptualization of health and disease, incorporating spiritual, physical and psycho-social factors [41]. In addition, the older adults had formed a stereotype that ‘minor illnesses/symptoms/pains’ were natural results of aging, and the inability to usual work was symptoms of ‘major diseases’ [42, 43]. This category of ‘minor illness’ and ‘major diseases’ was a compelling choice of older people because of the economic poverty and lack of medical resources in the past rural China. Despite suffering from symptoms of disease, the older adults in rural would not take any action until the illness had progressed to an immobilizing illness. In this sense, the boundary between health and disease had been blurred by their perception, thus leading to the misjudgment of health state. Thus, if healthcare providers accept individuals’ and communities’ unique definitions of health, their work will be more effective.

### Prioritization of relieving symptoms

Influenced by local cultures, environments and climates, Chinese ethnic groups combine the knowledge of traditional Chinese medicine with local herbs and foods and practice and develop their own local medicine for the prevention, diagnosis and treatment of illness and rehabilitation of health, which have established highly localized healthcare culture systems [44]. For example, the local medical system in southern China classifies constitutions and symptoms into ‘cool’ or ‘hot’ nature and applies prescriptions with herbs or foods antagonistic to these nature [20, 45]. These informal self-treating, which are often accessible, inexpensive and effective, have embedded in the memories of older people through word-of-mouth cases and have become widely accepted as alternative medical treatments [46]. Hence, the older adults in rural were prone to empirical self-treatment over seeking help from the modern healthcare system [7, 47, 48].

However, lots of older adults in rural suffer from one or more chronic diseases. Only when symptoms were not alleviated by self-treatment or the severity of symptoms gone beyond control did they turn to the healthcare system for formal treatment procedures [49–51]. Modern medicine features systemic explanations and remedies to disease onset and progression, including regular intake of medication, regular monitoring of health indicators, healthy lifestyles, dietary nutrition and so on in order to achieve recovery or optimal clinical outcomes. However, the treatment expectation of older people in rural was to remove symptoms but not cure diseases, which ran counter to the objective of modern medical treatment schemes. When they did seek services from professionals, they would selectively comply with the most convenient, effective, or cheapest medical suggestions in the wish to get back to their normal life as soon as possible. Not only did they want remain independent in their responsibility to sustain their families, but not overburden their families. The priority for older adults was symptomatic relief rather than preventing and curing diseases.

### ‘Controllable’ is the key to behavioral logic

While health perception and health practices under its guidance could explain the low utilization of healthcare services among older adults in rural, the underlying reason was that, when engaged in formal medical procedures, the established routines would be disturbed and the controlled later life would fall into a state of disorder. On the one hand, they were fearful of the extra social and financial costs incurred by their healthcare-seeking behavior [52, 53]. On the other hand, they worried about the need to spend a lot of foods or medicines of highly nutritious value to supplement the ‘energy damage’ caused by invasive examination and treatment, which might further exacerbate the hard-pressed life and aging body. Once healthcare-seeking had been practiced, they were likely to fall a passive position, where they passively accepted unpleasant results from the examinations and were forced to take various types of medical treatments. The life was put into uncertainty. These findings revealed significant discrepancies between the health recommendations and actual practices of older people in rural areas, the explanation might be insufficient health literacy. Health literacy levels in China is 23.15%, and it is much lower in rural areas than in urban areas [54]. Improving health literacy requires a culturally adaptable health education. Given that more and more people are seeking health information online. It is recommended that health education activities in rural areas be conducted online and offline to spread health knowledge.

### ‘Two-way’ accessibility of medical resources

In summary, older adults have access to affordable health services close to their homes [14]. However, the density of medical resource distribution has not led to significant increase in the utilization of health services [29, 55]. Therefore, this study proposed in the concept of ‘two-way’ accessibility of rural health services. ‘Two-way’ accessibility of medical resources was the result of the researcher’s findings from the graphs and interviews: rural older people had accessible and affordable health services and plenty of time to make use of these, but in fact they failed to act on their health needs, and blind to health services and made these ‘inaccessible’.

### Strengths and limitations

Our study adopts the thematic analysis method of anthropology to analyze the behavioral logic of medical practice of the old Chinese adults in rural from the thematic perspective. A community empowerment environment for participants was created by PRA, which make the data collects from the bottom up. Our study had some limitations. All the participants in this study came from a rural area in southern China. As with all qualitative research, the findings in this study could not be generalized. Furthermore, the participants in this study were all suffering from more than one chronic disease, and the older adults with serious diseases were not included with their medical practice experience being different.

## Conclusion

Our study showed that the inherent logic behind the refusal of healthcare utilization among the older adults in rural China was not simply the evasion of healthcare services. In effect, the underlying reason was that they were influenced by a long-established health perception and its derived health practices. From the perspective of their health perception, they were not without healthcare-seeking behaviors, they were just more likely to adopt empirical and informal self-treating. On the other hand, the risk of orderly and controlled life to be broken down by medical interventions was the underlying reason why they dismissed healthcare-seeking behaviors to maintain the status quo. As a result, they might miss the best time for the treatment of their illness, which would worsen their illness worse.

It is clear that rural health-care reform that focuses on the allocation of health resources or the design of insurance seems ineffective. Patients’ health concepts are critical in predicting their health behaviors and their adherence to medical programs. To a certain degree, health is subjective, its interpretation differing considerably from one socio-cultural environment to another. Understanding the defining attributes of health can support better approaches to health care and health promotion, particularly among rural subcultures such as farmers. Therefore, the health care reform in rural should also consider the power of culture, and combine with the local culture, so that the reform will be more effective.

### Electronic supplementary material

Below is the link to the electronic supplementary material.

Appendix 1: COREQ checklist

Appendix 2: Tools for PRA

## Data Availability

The datasets generated and/or analyzed during the current study are not publicly available in the interest of participant privacy and confidentiality but are available from the corresponding author on reasonable request.

## Abbreviations

PRA: Participatory rural appraisal
NCMS: The New Rural Cooperative Medical Scheme
NHC: The National Health Commission
COREQ: The Consolidated Criteria for Reporting Qualitative study checklist
