# Supplementary material for: The action logic of the older adults about health-seeking in South Rural China
Source: BMC Public Health. 2023 Dec 12;23:2487. doi: 10.1186/s12889-023-17314-y (PMC10714459; doi:10.1186/s12889-023-17314-y)
Supplement: Supplementary file 1 — Appendix 1: COREQ checklist [file 12889_2023_17314_MOESM1_ESM.pdf]

Appendix 1. COREQ checklist

Reporting Guideline Checklist

（Consolidated criteria for reporting qualitative studies, COREQ）

No Item	Guide questions/description	
**Domain 1: Research team and reflexivity**		
**Personal Characteristics**		
1. Interviewer/facilitator	Which author/s conducted the interview or focus group?	All authors
2. Credentials	What were the researcher's credentials? E.g. PhD, MD	Jianqiang Lin- PhD<br>Dan Yang- BD<br>Xinyu Zhao-PhD<br>Liqiong Xie-BD<br>Kun Xiong-MD<br>Lei Hu- BD<br>Yue Xu-PhD<br>ShanShan Yu-PhD<br>Wenyong Huang-PhD<br>Ni Gong- PhD<br>Xiaoling Liang- PhD
3. Occupation	What was their occupation at the time of the study?	Jianqiang Lin- Doctor<br>Dan Yang-Postgraduate student<br>Xinyu Zhao-Doctor<br>Liqiong Xie-Research assistant<br>Kun Xiong-Doctor<br>Lei Hu-Postgraduate student<br>Yue Xu-Doctor<br>ShanShan Yu-Doctor<br>Wenyong Huang-Doctor<br>Ni Gong- Associate professor<br>Xiaoling Liang-Doctor
4. Gender	Was the researcher male or female?	Jianqiang Lin- Male<br>Dan Yang- Female<br>Xinyu Zhao-Female<br>Liqiong Xie-Female<br>Kun Xiong-Male<br>Lei Hu- Female<br>Yue Xu-Male<br>ShanShan Yu-Female<br>Wenyong Huang-Male<br>Ni Gong- Male<br>Xiaoling Liang-Female
5. Experience and training	What experience or training did the researcher have?	Jianqiang Lin- Completed some qualitative studies

		Dan Yang- Community nursing internship experience<br>Xinyu Zhao- Received systematic training<br>Liqiong Xie- Received systematic training<br>Kun Xiong- Received systematic training<br>Lei Hu- Community nursing internship experience<br>Yue Xu- Received systematic training<br>ShanShan Yu- Received systematic training<br>Wenyong Huang- Received systematic training<br>Ni Gong- Completed a large number of qualitative studies<br>Xiaoling Liang- Received systematic training
**Relationship with participants**		
6. Relationship established	Was a relationship established prior to study commencement?	No
7. Participant knowledge of the interviewer	What did the participants know about the researcher? e.g. personal goals, reasons for doing the research	The participants know about reasons for doing the research, and the purpose of the research.
8. Interviewer characteristics	What characteristics were reported about the interviewer/facilitator? e.g. Bias, assumptions, reasons and interests in the research topic	The interviewer wanted to explore reasons that have discouraged health-seeking behavior among older adults in rural China.
**Domain 2: study design**		
**Theoretical framework**		
9. Methodological orientation and Theory	What methodological orientation was stated to underpin the study? e.g. grounded theory, discourse analysis, ethnography, phenomenology, content analysis	content analysis
**Participant selection**		

10. Sampling	How were participants selected? e.g. purposive, convenience, consecutive, snowball	Purposive sampling
11. Method of approach	How were participants approached? e.g. face-to-face, telephone, mail, email	Face-to-face
12. Sample size	How many participants were in the study?	108
13. Non-participation	How many people refused to participate or dropped out? Reasons?	None
**Setting**		
14. Setting of data collection	Where was the data collected? e.g. home, clinic, workplace	Participants' home
15. Presence of non-participants	Was anyone else present besides the participants and researchers?	None
16. Description of sample	What are the important characteristics of the sample? e.g. demographic data, date	Table 1.
**Data collection**		
17. Interview guide	Were questions, prompts, guides provided by the authors? Was it pilot tested?	Yes<br>Yes
18. Repeat interviews	Were repeat interviews carried out? If yes, how many?	No
19. Audio/visual recording	Did the research use audio or visual recording to collect the data?	The research used audio recording to collect the data.
20. Field notes	Were field notes made during and/or after the interview or focus group?	Field notes made during the interview
21. Duration	What was the duration of the interviews or focus group?	From 20 to 50 minutes
22. Data saturation	Was data saturation discussed?	Yes
23. Transcripts returned	Were transcripts returned to participants for comment and/or correction?	No
**Domain 3: analysis and findings**		
**Data analysis**		
24. Number of data coders	How many data coders coded the data?	Eleven
25. Description of the coding tree	Did authors provide a description of the coding tree?	No
26. Derivation of themes	Were themes identified in	Derived from the data

	advance or derived from the data?	
27. Software	What software, if applicable, was used to manage the data?	NVivo 11
28. Participant checking	Did participants provide feedback on the findings?	No
**Reporting**		
29. Quotations presented	Were participant quotations presented to illustrate the themes / findings? Was each quotation identified? e.g. participant number	Yes
30. Data and findings consistent	Was there consistency between the data presented and the findings?	Yes
31. Clarity of major themes	Were major themes clearly presented in the findings?	Yes
32. Clarity of minor themes	Is there a description of diverse cases or discussion of minor themes?	Yes
