# Supplementary material for: The action logic of the older adults about health-seeking in South Rural China
Source: BMC Public Health. 2023 Dec 12;23:2487. doi: 10.1186/s12889-023-17314-y (PMC10714459; doi:10.1186/s12889-023-17314-y)
Supplement: Supplementary file 2 — Appendix 2: Tools for PRA [file 12889_2023_17314_MOESM2_ESM.pdf]

Appendix 2 Tools for PRA

1. Social and Resource Mapping

Please draw on this piece of paper where you visit regularly or have significant meaning (Azimuth, approximate distance).

2. Daily Schedule

Please write down your daily activities at the corresponding time points in the daily schedule.

3. Interview Guide for the old adults in rural areas

(1) How is your health condition?

(2) What do you think about your health problem?

(3) How do you manage your health problem specifically?

(4) How do you deal with it when you don't feel well?

(5) Do you have any special treatment?

(6) Do you find these treatments effective? Why?

(7) What do you think health is?

(8) How do you keep healthy?

(9) Have you ever had a free physical examination? Which checks?

(10) Do you know what items are included in the basic medical services for older adults?

(11) Do you have any physical items you don't want to? Why? (Introduce the physical examination according to the actual situation)
